# Diagnostic and Therapeutic Considerations in Concurrent Plasma Cell Dyscrasia and Amyloidosis

**DOI:** 10.5339/qmj.2022.44

**Published:** 2022-09-15

**Authors:** Muhamed Baljevic

**Affiliations:** Vanderbilt-Ingram Cancer Center, Vanderbilt University Medical Center, Nashville, TN, The United States of America E-mail: muhamed.baljevic@vumc.org

**Keywords:** amyloidosis, diagnosis, plasma cell dyscrasia

Amyloidosis is a systemic disorder of deposition of more than 30 different types of misfolded precursor proteins in extracellular tissues, leading to variety of organ dysfunctions and subsequent clinical presentations. Cardiac involvement usually results in restrictive cardiomyopathy and diastolic dysfunction leading to congestive heart failure, although conduction system defects and arrhythmias are also very common^1^. However, as the two most common forms of amyloidosis, immunoglobulin light chain (AL) and wild type transthyretin (ATTRwt) amyloidosis do coexist, very careful and stepwise approach needs to be employed in diagnostically evaluating patients who have evidence of amyloid organ deposition with concurrent plasma cell dyscrasia (PCD)^2^. The report that describes cardiac amyloid as a presenting feature of multiple myeloma (MM) by Velayutham et al., unfortunately lacks the necessary rigor in verifying the exact etiology of cardiac amyloidosis, and further misidentifies it as a presenting feature of newly diagnosed MM^3^. This can critically influence the choice of appropriate starting therapy.

Although the 2003 International Myeloma Working Group (IMWG) criteria included non- hypercalcemia, renal insufficiency, anemia, and bone lesions (CRAB) end-organ damage, such as hyperviscosity, AL amyloidosis, and recurrent bacterial infections as sufficient criteria for new diagnosis of MM, the 2014 update regards systemic AL amyloidosis as a distinct PCD, and amyloid presence in a patient with monoclonal gammopathy does not automatically suggest MM nor AL amyloidosis^2,4^. Furthermore, IMWG 2014 guidelines do not recommend the use of non-CRAB criteria for the initiation of treatment. While a concurrent presence of MM or increased bone marrow plasmacytosis is very much possible in AL Amyloidosis, and is well described as a high-risk feature in these patients^5^, institution of therapy without the adequate confirmation of amyloid subtype, especially in a patient that otherwise lacks clear myeloma defining events represents a significant clinical risk of mistreatment and potential harm to the patient.

We wish to highlight the vital importance of accurate fibril subtyping in patients with amyloidosis, irrespective of the presence of concurrent PCD. Before instituting PCD-directed therapy, it is essential to establish the evidence that the amyloid is immunoglobulin (heavy or light) chain-related via direct examination of the amyloid, ideally using liquid chromatography and mass spectrometry or immunoelectron microscopy^6^ (Figure 1). In circumstances of limited resources, immunohistochemistry including immunofluorescence, performed by a highly specialized pathologist combined with clinical examination and genotyping leads to a high accuracy of amyloidosis classification, and can be considered acceptable^7^. The case by Velayutham et al.,^3^ made no reference to such efforts, and it remains unclear if the cardiac amyloidosis was in fact in the context of AL or some other form of amyloidosis.

We now have treatment for both the ATTRwt and hereditary forms of transthyretin amyloid cardiomyopathy, which are associated with reductions in all-cause mortality, cardiovascular-related hospitalizations as well as lower decline in functional capacity and quality of life^10^.

Cardiomyopathy represents the leading cause of death in AL amyloidosis, and is unfortunately not rare: 60% of patients have clinically significant cardiac involvement as a presenting manifestation^1^. Lastly, it should be noted that while lenalidomide therapy has been studied in newly diagnosed AL Amyloidosis, it may be challenging in terms of toxicity, especially in those with renal and cardiac dysfunction. Hence, patients with cardiac amyloidosis should be carefully monitored while on immunomodulatory drugs^8,9^.

Current standard for the treatment of systemic light chain amyloidosis includes combination therapy with daratumumab, bortezomib, cyclophosphamide and dexamethasone^11^.

## Figures and Tables

**Figure 1.**
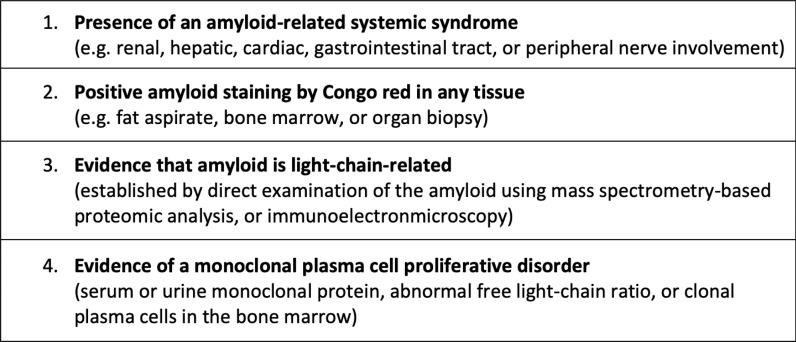
Required (all four) diagnostic criteria for systemic light chain amyloidosis^4^

## References

[1] Mankad AK, Sesay I, Shah KB (2017). Light-chain cardiac amyloidosis. Curr Probl Cancer.

[2] Sidiqi MH, Dasari S, McPhail ED (2019). Monoclonal gammopathy plus positive amyloid biopsy does not always equal AL amyloidosis. Am J Hematol.

[3] Velayutham R, Parale C, Sukumaran SK, Anantharaj A (2022). Cardiac Amyloid as a presenting feature of multiple Myeloma. QJM.

[4] Rajkumar SV, Dimopoulos MA, Palumbo A (2014). International Myeloma Working Group updated criteria for the diagnosis of multiple myeloma. The Lancet Oncology.

[5] Kourelis TV, Kumar SK, Gertz MA (2013). Coexistent multiple myeloma or increased bone marrow plasma cells define equally high-risk populations in patients with immunoglobulin light chain amyloidosis. J Clin Oncol.

[6] Bianchi G, Kumar S (2020). Systemic Amyloidosis Due to Clonal Plasma Cell Diseases. Hematol Oncol Clin North Am.

[7] Schonland SO, Hegenbart U, Bochtler T (2012). Immunohistochemistry in the classification of systemic forms of amyloidosis: a systematic investigation of 117 patients. Blood.

[8] https://www.nccn.org/professionals/physician_gls/pdf/amyloidosis.pdf.

[9] Kastritis E, Dialoupi I, Gavriatopoulou M (2019). Primary treatment of light-chain amyloidosis with bortezomib, lenalidomide, and dexamethasone. Blood Adv.

[10] Maurer MS, Schwartz JH, Gundapaneni B (2018). Tafamidis Treatment for Patients with Transthyretin Amyloid Cardiomyopathy. N Engl J Med.

[11] Kastritis E, Palladini G, Minnema MC (2021). N Engl J Med.

